# Proteomic screening of molecular targets of crocin

**DOI:** 10.1186/2008-2231-22-5

**Published:** 2014-01-06

**Authors:** Hossein Hosseinzadeh, Soghra Mehri, Ali Heshmati, Mohammad Ramezani, Amirhossein Sahebkar, Khalil Abnous

**Affiliations:** 1 Pharmaceutical Research Center, Department of Pharmacodynamics and Toxicology, School of Pharmacy, Mashhad University of Medical Sciences, Mashhad, Iran; 2 School of Pharmacy, Mashhad University of Medical Sciences, Mashhad, Iran; 3 Pharmaceutical and Biotechnology Research Centers, School of Pharmacy, Mashhad University of Medical Sciences, Mashhad, Iran; 4 Biotechnology Research Center, Mashhad University of Medical Sciences, Mashhad, Iran; 5 Pharmaceutical Research Center, Department of Medicinal Chemistry, School of Pharmacy, Mashhad University of Medical Sciences, 91775-1365 Mashhad, Iran

**Keywords:** *Crocus sativus* L, Crocin, Target Deconvolution, Affinity chromatography, Target deconvolution, Electrophoresis

## Abstract

**Background:**

Traditional drug discovery approaches are mainly relied on the observed phenotypic changes following administration of a plant extract, drug candidate or natural product. Recently, target-based approaches are becoming more popular. The present study aimed to identify the cellular targets of crocin, the bioactive dietary carotenoid present in saffron, using an affinity-based method.

**Methods:**

Heart, kidney and brain tissues of BALB/c mice were homogenized and extracted for the experiments. Target deconvolution was carried out by first passing cell lysate through an affinity column prepared by covalently attaching crocin to agarose beads. Isolated proteins were separated on a 2D gel, trypsinized *in situ* and identified by MALDI-TOF/TOF mass spectrometry. MASCOT search engine was used to analyze Mass Data.

**Results:**

Part of proteome that physically interacts with crocin was found to consist of beta-actin-like protein 2, cytochrome b-c1 complex subunit 1, ATP synthase subunit beta, tubulin beta-3 chain, tubulin beta-6 chain, 14-3-3 protein beta/alpha, V-type proton ATPase catalytic subunitA, 60 kDa heat shock protein, creatine kinase b-type, peroxiredoxin-2, cytochrome b-c1 complex subunit 2, acetyl-coA acetyltransferase, cytochrome c1, proteasome subunit alpha type-6 and proteasome subunit alpha type-4.

**Conclusion:**

The present findings revealed that crocin physically binds to a wide range of cellular proteins such as structural proteins, membrane transporters, and enzymes involved in ATP and redox homeostasis and signal transduction.

## Introduction

Dried stigma of *Crocus sativus* L. (Iridaceae), called saffron, is a widely used dietary spice and food colorant [1]. Aside from culinary purposes, saffron has been used in several traditional systems of medicine for the treatment of numerous diseases such as cough, colic, insomnia, chronic uterine hemorrhage, cardiovascular disorders and tumors [2].

**Figure 1:**
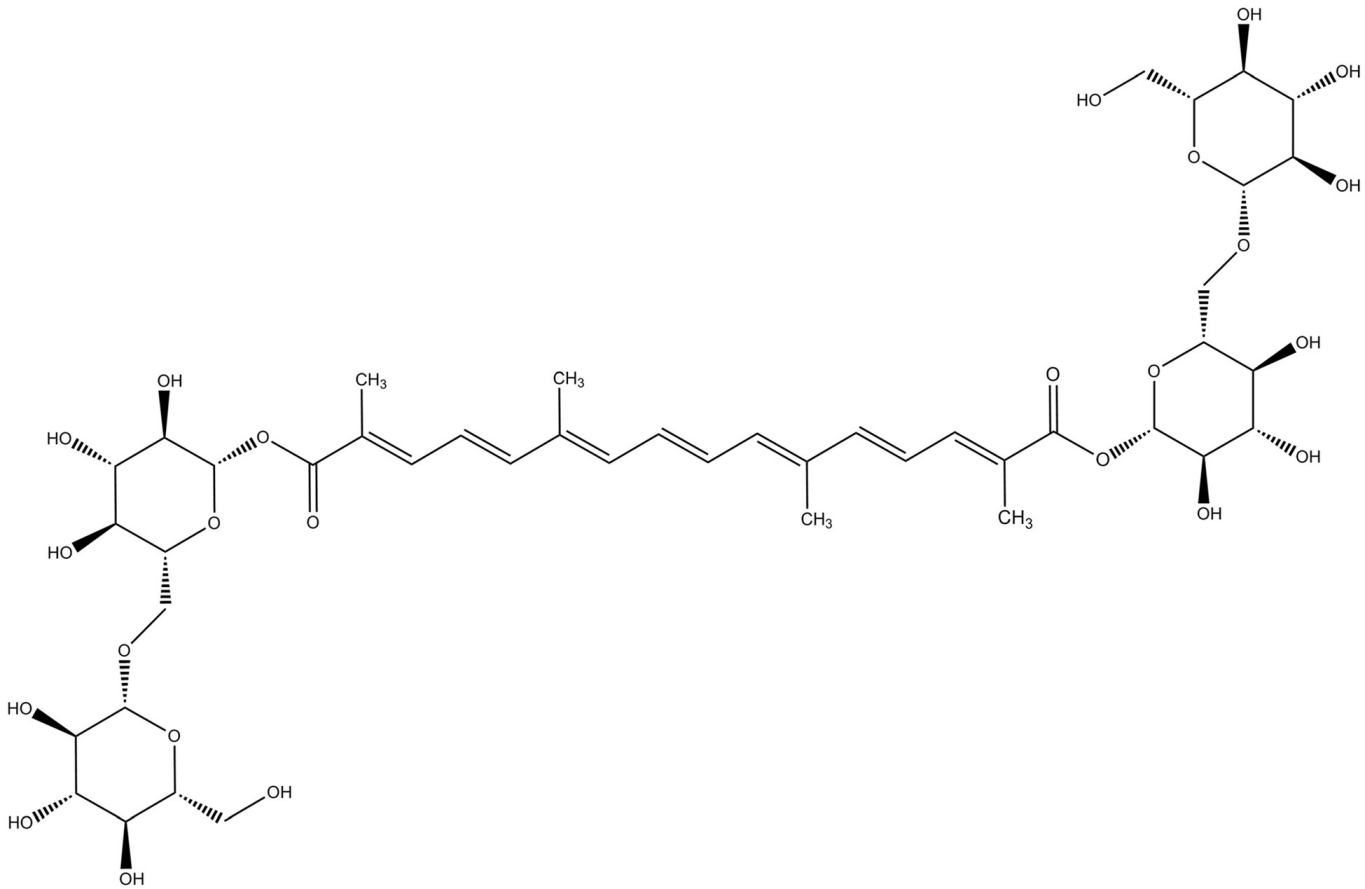
Chemical structure of crocin.

Crocin (Figure 1) is a bioactive carotenoid present in *C. sativus*, and is responsible for the golden-yellow color of saffron [3]. Modern scientific investigations have unveiled several interesting pharmacological activities for crocin including, but not limited to, antitumor [2], radical scavenging [4], antidepressant [5] and memory-enhancing effects [6]. In addition, crocin has been shown to possess high antioxidant and anti-proliferative capacities in both *in-vitro* and *in-vivo* conditions [7-11]. Yet, it must be taken into accurate account that the anti-tumor properties of crocin, like some other phytochemicals, are likely to be independent of the well-known antioxidant actions. The notion of antioxidants as potential anti-cancer agents has recently been questioned due to some observations on the lack of efficacy of antioxidant therapy in the treatment of cancer [12]. Besides, it is known that some chemotherapy agents exert their cytotoxic effects via induction of oxidative stress [13]. Finally, the fact that crocin induces apoptosis in cancerous cells – a phenomonen usually associated with increased generationof free radicals – is another proof for the lack of association between antioxidant and anti-cancer properties of this compound [14,15]. It has been hypothesized that phytochemicals with dual antioxidant/anti-cancer properties may exert the latter effect via epigenetic mechanisms including promotion of DNA demethylation, histone modification and RNA interference [16,17]. However, unraveling the mechanisms underlying the antioxidant and anti-cancer properties of crocin is warranted for further clarification in this context. Moreover, dose-effect studies need to be undertaken in order to identify optimal doses at which antioxidant and anti-cancer effects of crocin predominate.

In spite of proven benefits, molecular mechanisms that account for the pharmacological effects of crocin have remained largely unknown. However, several lines of evidence have demonstrated that phytochemicals promote their biological and health promoting effects through interaction with a variety of structural and functional proteins [18].

Traditional approach toward drug discovery has been mainly based on the observation of a phenotypic change following application of a plant extract, drug candidate or a natural product. Recently, target-based approaches are becoming more popular. The identification of target proteins for newly developed drugs or natural products is regarded as "target deconvolution" [19,20]. Such an identification of the potential targets of a small pharmacologically active molecule helps elucidating the primary mechanism of action, prediction of side effects and unwanted off-target interactions, and finding new potential therapeutic effects.

The present study hypothesized that pharmacological activities of crocin depend, at least in part, on its physical interaction with cellular proteins. Hence, part of the cellular proteome that binds to crocin was isolated from tissue lysates using affinity chromatography and subjected to mass specterometry (MS)-based proteomic analysis to identify the potential molecular targets of this phytochemical.

## Material and methods

### Crocin extraction and purification

Stigmas of *C. sativus* L. were collected from Ghaen, Khorasan province, Northeast of Iran, and provided by Novin Saffron Co. (Mashhad, Iran). Analysis and quality control of samples was conducted in accordance to the ISO/TS 3632–2 standards. Extraction and purification of crocin from saffron was carried out as previously described by Hadizadeh and colleagues [21].

### Animals

Twelve BALB/c mice (20–25 g) were killed by decapitation. Heart, kidney and brain tissues of mice were collected and washed using 0.9% normal saline solution. Tissues were immediately frozen in liquid nitrogen and transferred to -80°C until use. All animal experiments were carried out in accordance with the acts of the Mashhad University of Medical Sciences Ethics Committee (code 87772).

### Preparation of tissue extracts

Each sample (200–400 mg) was homogenized 1:5 (w:v) in extraction buffer containing 50 mM Tris (pH 7.4), 2 mM EGTA, 2 mM EDTA, 2 mM Na_3_VO_4,_, 1% Triton X-100 and 10 mM 2-mercaptoethanol with further addition of a few crystals of the protease inhibitor, phenylmethylsulfonyl fluoride (PMSF) immediately before homogenization of tissue. Samples were homogenized using a Polytron Homogenizer (Kinematica, Switzerland) for 10 sec followed by sonication (UP100H, Hielscher) for 40 sec and centrifugation (Hettich Universal 320R, Germany) at 25,000 g for 10 min at 4°C. The supernatant was then removed and stored on ice. Protein contents were measured using Bradford protein assay (BioRad). The protein contents of all samples were adjusted to 2 mg/mL.

### Preparation of crocin-resin conjugate

Crocin affinity matrix was prepared using pharmaLink Kit (Pierce) according to the manufacturer’s instructions. Briefly, agarose beads containing immobilized diaminodipropylamine (DADPA) were equilibrated in 4 mL coupling buffer (0.1 M MES, 0.15 M NaCl, pH 4.7). Crocin (100 mg) was dissolved in 2 mL of coupling buffer and transferred to the aforementioned resin slurry. Coupling reaction was started by adding 200 μL of coupling reagent (37% formaldehyde solution) to the resin/crocin mixture. Reaction mixture was incubated for 72 h in 50°C. To remove free crocin, resin slurry was transferred to a column and washed 12 times each time with 2 mL of wash buffer (0.1 M Tris, pH 8.0). Flowthrough fractions were collected and pooled. Quantity of free crocin was calculated by measuring absorbance of pooled flowthrough fractions at 441.6 nm using visible spectroscopy (CECIL 9000 Series). Efficiency of crocin conjugation to resin was calculated using the following equation:

$$\mathrm{\%Resin}-\mathrm{conjugated}\;\mathrm{crocin}=\frac{\mathrm{mg}\;\left(\mathrm{totatl}\;\mathrm{crocin}\right)-\mathrm{mg}\;\left(\mathrm{free}\;\mathrm{crocin}\right)}{\mathrm{mg}\;\left(\mathrm{total}\;\mathrm{crocin}\right)}$$

### Affinity chromatography

Affinity chromatography was performed to isolate molecular targets of crocin. Briefly, both controlss (affinity column without crocin) and affinity column were equilibrated in binding buffer [50 mM Tris (pH 7.4), 2 mM EGTA, 2 mM EDTA, 2 mM Na_3_VO_4,_, 1% Triton X-100, and 10 mM 2-ME]. Tissue extracts were incubated with control column resin for 30 min at 4°C. After a brief centrifugation at 1000 g, supernatants were transferred to the affinity column. Following incubation for 30 min at 4°C, affinity column was washed 4 times each time with 2 mL of binding buffer. Crocin target proteins were eluted using 2 mL of 2 M NaCl in binding buffer. The elution was repeated 3 more times and fractions were pooled. The presence of proteins in fractions was tested using Bradford protein assay kit (BioRad). The pooled fractions were dialyzed at a 2000 Da cut-off to remove electrolytes. To concentrate target proteins, samples were freeze-dried and stored at -20°C until use.

### 2D gel electrophoresis

Freeze-dried samples were dissolved to a final concentration of 125 μg/125 μL in rehydration buffer containing 6 M urea, 2 M thiourea, 2% (3-[(3-Cholamidopropyl)-dimethylammonio]-1-propane sulfonate) (CHAPS), 50 mM dithiothreitol (DTT) and 20% Bio-Lyte (BioRad). Non-linear immobilized pH gradients (IPGs) (pH 3–10; BioRad) were used to separate crocin target proteins based on their isoelectric point [1]. For passive rehydration, IPGs and protein solutions were incubated at room temperature for 12 h. Isoelectric focusing was performed using PROTEAN IEF cell (BioRad) at 4000 V for 11 h. After isoelectric focusing, IPGs were incubated in equilibration buffer [375 mM Tris (pH 8.8), 6 M Urea, 2.5% SDS and 30% glycerol] for 20 min. Then, IPGs were placed on top of 12% sodium dodecyl sulfate-polyacrylamide gel electrophoresis (SDS-PAGE) and sealed with heated agarose solution (25 mM Tris (pH 8.8), 84 mM glycine, 0.5% agarose, 0.1% SDS and small amount of tracking dye bromophenol blue). Electrophoresis was performed for 80 min at 120 V. Gels were silver stained and protein spots were excised and collected in microtubes.

### In-gel digestion

Gel slices were incubated in destaining buffer (50% MeOH, 5% acetic acid) overnight at room temperature. Destaining was repeated with fresh buffer for 2 more h. Gel slices were dehydrated in acetonitrile for 30 min and dried in vacufuge. Afterwards, gels were covered with reducing buffer (1.5 mg/mL DTT in 100 mM ammonium bicarbonate) for 1 h. Protein alkylation was performed by incubation of gel slices in 100 μL of 10 mg/mL iodoacetamide in 100 mM ammonium bicarbonate for 30 min at room temperature. Gel slices were washed using 0.5 mL of 100 mM ammonium bicarbonate followed by dehydration using acetonitrile and drying in vacufuge. Then, 50 μL of 20 μg/mL trypsin was added to each gel slice and incubated overnight at 4°C. Peptides were extracted in 3 steps by adding 100 μL of 100 mM ammonium bicarbonate, 100 μL extraction solution (50% acetonitrile and 5% formic acid) and finally 150 μL extraction solution. Samples were dried down to a final volume of 15 μL in vacufuge and desalted using ZipTip® μC-18 (Millipore). Eluted samples were stored at -20°C until use.

### Mass analysis

Mass analysis was performed at Genome Research Centre at the University of Hong Kong using a 4800 MALDI-TOF/TOF analyzer (ABI). In-house MASCOT search engine was used to analyze Mass Data. Data were BLASTed against both NCBInr and SwissProt databases. MASCOT parameters were set as follow: Taxonomy: mouse, fixed modification: carbamidomethyl (C), variable modification: oxidation (M), MS/MS fragment tolerance: 0.2 Da, precursor tolerance: 75 ppm, peptide charge: +1, monoisotopic. MASCOT cut-off scores were set to 30. Only the peptides ranked first with *p*-values smaller than 0.05 were accepted.

## Results

**Figure 2:**
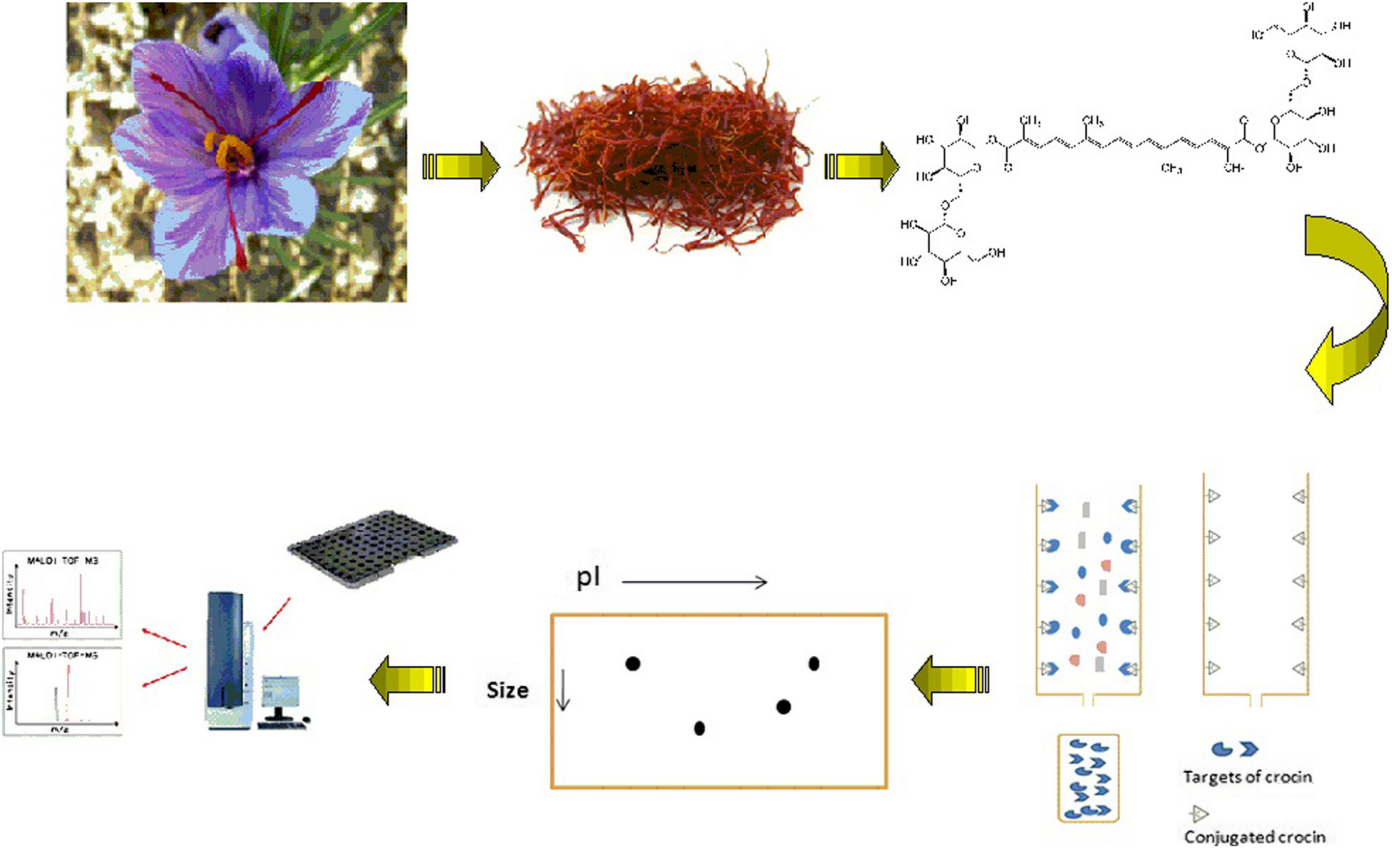
A schematic summary of study design and methodology.

Affinity chromatography was performed to find cellular targets of crocin in different organs. There are two types of interactions between stationary phase and cellular proteins in affinity chromatography: specific interaction between crocin and its targets or unspecific binding of proteins to other parts of stationary phase such as agarose beads. To reduce unspecific binding of non-target proteins, tissue extracts were incubated with control agarose beads. Unbound proteins were incubated with crocin-resin beads. Target proteins were eluted using 2 M NaCl in binding buffer and subjected to 2D gel electrophoresis. After in-gel digestion of protein spots, MALDI TOF/TOF was employed for their identification. Mass data were analyzed using MASCOT (Figure 2; Additional file 1).

### Crocin-resin conjugation

**Figure 3:**
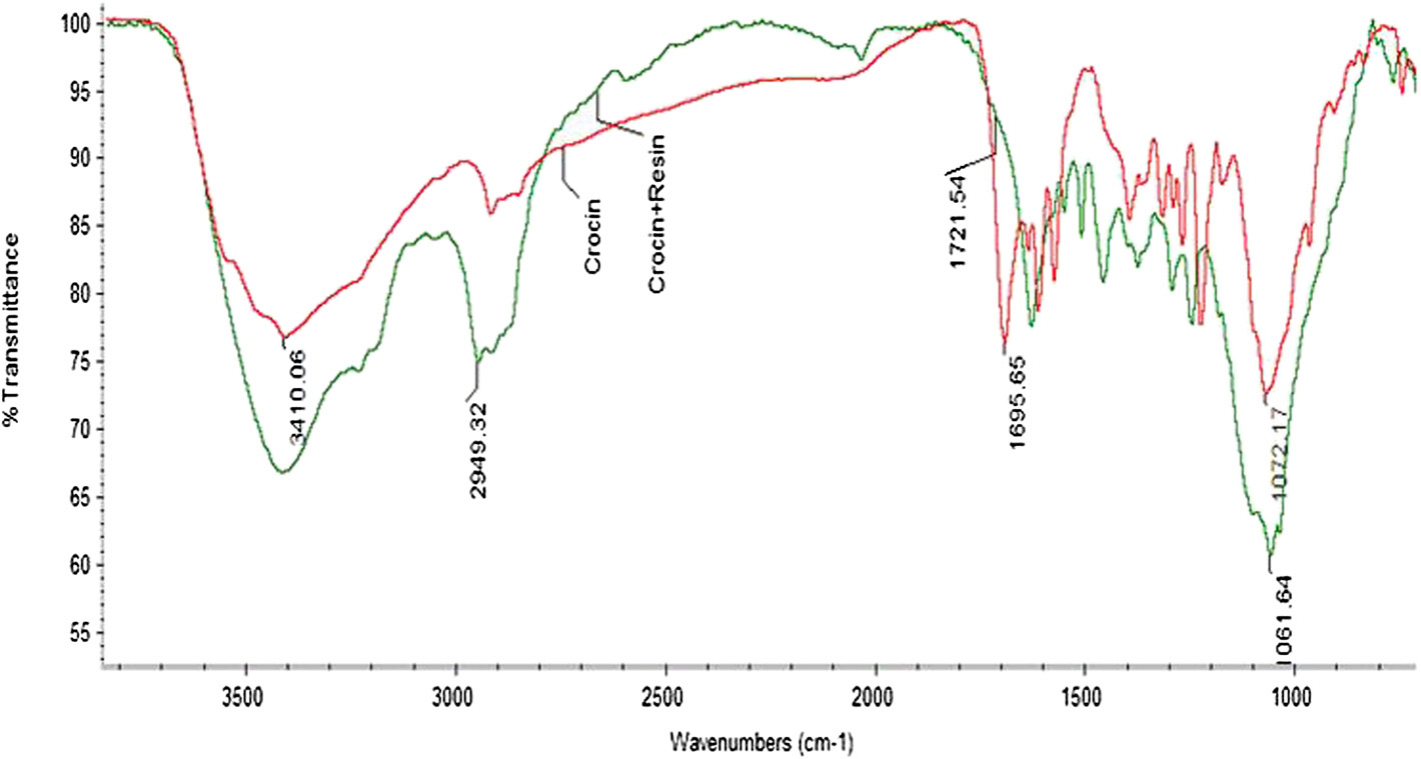
FT-IR spectrum of crocin (red) and crocin-resin conjugate (green).

Crocin was covalently attached to diaminodipropylamine side chain of agarose beads using Mannich reaction. Briefly, formaldehyde reacts with primary amino group to produce highly reactive iminium group. This group can react with active hydrogen on hydroxyl groups of sugar residues on crocin. Yield of crocin conjugation to agarose beads was calculated to be 70%. In FT-IR spectrum of crocin-resin conjugate, hydroxyl groups (-OH) of crocin glycosides were observed at 3410.06 cm^-1^ which is identical with that of pure crocin (Figure 3). Besides other peaks in the crocin-resin conjugate were overlapped with those of pure crocin, suggesting the presence of crocin with its functional groups within the conjugate.

### Target proteins of crocin in kidney

**Figure 4:**
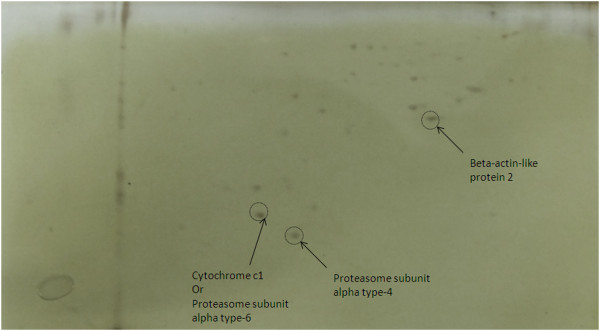
**2D gel electrophoresis of crocin targets in kidney extract.** Spots were identified as Beta-actin-like protein 2, cytochrome c1, proteasome subunit alpha type-6 and proteasome subunit alpha type-4.

Beta-actin-like protein 2, cytochrome c1, proteasome subunit alpha type-6 and proteasome subunit alpha type-4 were identified as cellular targets of crocin in kidney (Figure 4 and Table 1).

**Table 1:** Target proteins of crocin as identified by MALDI-TOF/TOF and MASCOT

	**Protein name**	**Protein score**	**Protein score C.I.%**	**MW/pI**	**Accession number**
1	Acetyl-CoA acetyltransferase	81	99	45KDa/8.7	Gi 21450129
2	V-type proton ATPase catalytic subunitA	113	100	68.6KDa/5.42	P50516.2
3	Proteasome subunit alpha type-4	159	100	30/KDa/7.59	Q9R1P0.1
4	14-3-3 protein beta/alpha	160	100	28KDa/4.77	Q9CQV8.3
5	Tubulin beta-6 chain	169	100	50.5KDa/4.8	Q922F4.1
6	Proteasome subunit alpha type-6	170	100	28KDa/6.34	Q9QUM9.1
7	Beta-actin-like protein 2	215	100	42KDa/5.3	Q8BFZ3
8	Tubulin beta-3 chain	258	100	51KDa/4.82	Q9ERD7.1
9	Cytochrome b-c1 complex subunit 1	265	100	53KDa/5.75	Q9CZ13.1
10	Cytochrome c1	277	100	35.5KDa/9.24	Q9DOM3.1
11	Peroxiredoxin-2	283	100	22KDa/5.2	Q61171.3
12	Cytochrome b-c1 complex subunit 2	323	100	48KDa/9.26	Q9DB77.1
13	60 kDa heat shock protein	348	100	61KDa/6.33	Gi 247242
14	Creatine kinase B-type	388	100	43KDa/5.4	Q04447.1
15	ATP synthase subunit beta	485	100	56KDa/5.19	P56480.2

### Target proteins of crocin in heart

**Figure 5:**
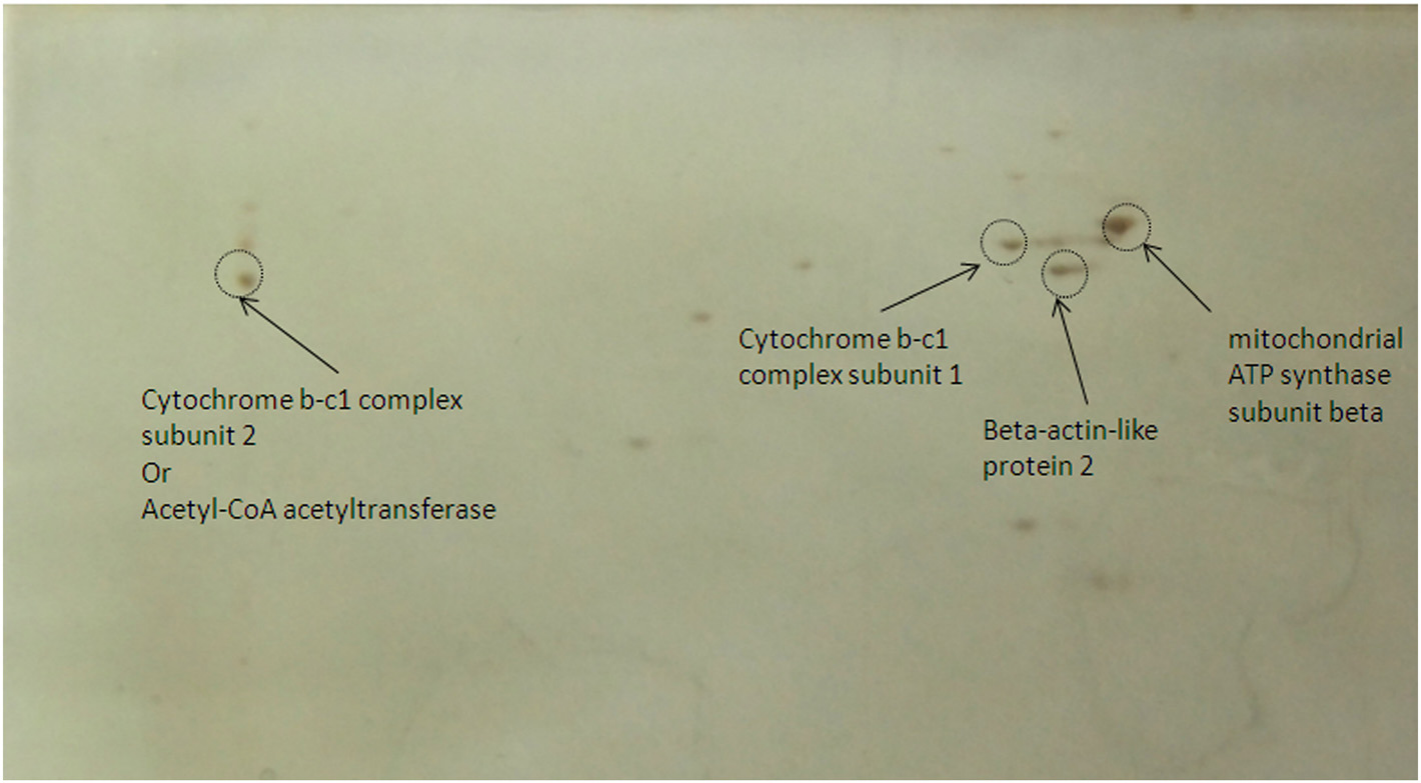
**2D gel electrophoresis of crocin targets in heart extract.** Spots were identified as mitochondrial ATP synthase subunit beta, beta-actin-like protein 2, cytochrome b-c1 complex subunit 1 and subunit 2 and *acetyl-CoA acetyltransferase.*

Data indicated that crocin binds to mitochondrial ATP synthase subunit beta, beta-actin-like protein 2, cytochrome b-c1 complex subunit 1 and subunit 2, and acetyl-CoA acetyltransferase in heart (Figure 5 and Table 1).

### Target proteins of crocin in Brain

**Figure 6:**
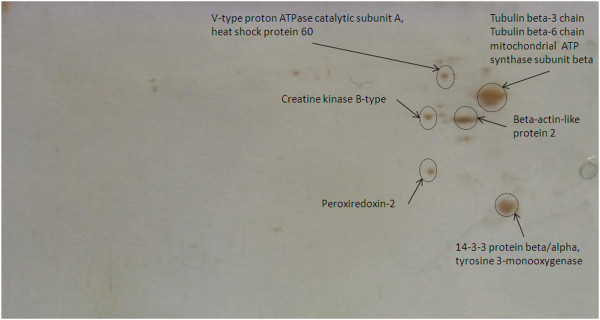
**2D gel electrophoresis of crocin targets in brain extract.** Spots were identified as tubulin beta-3 chain, tubulin beta-6 chain, mitochondrial ATP synthase, beta-actin-like protein 2, 14-3-3 protein beta/alpha, tyrosine 3-monooxygenase, V-type proton ATPase catalytic subunit A, 60 kDa heat shock protein, creatine kinase B-type and peroxiredoxin-2.

Target proteins of crocin in brain were identified as tubulin beta-3 chain, tubulin beta-6 chain, mitochondrial ATP synthase, beta-actin-like protein 2, 14-3-3 protein beta/alpha, tyrosine 3-monooxygenase, V-type proton ATPase catalytic subunit A, 60 kDa heat shock protein, creatine kinase B-type and peroxiredoxin-2 (Figure 6 and Table 1).

## Discussion

Saffron has been mentioned in the folk medicine to have a warm and dry temperament [22]. This plant is endowed with a variety of health benefits including exhilarant, liver tonic and deobstruent, aphrodisiac, labour-inducing, emmenagogue, digestive, hypnotic, cardioprotective, anti-inflammatory and bronchodilatory properties [22,23]. Interestingly, most of these traditional uses are consistent with the findings of modern pharmacological research [23].

In the present study, affinity purification was exploited to identify cellular proteins that could physically interact with crocin. This technique has a distinct superiority to other deconvolution methods such as biochemical fractionation, phage display and expression cloning as it is more relevant to be used with crude cellular samples in which the proteins are in their intact biological form [24].

Drugs are normally discovered based on their ability to show a certain desired biological outcome. For instance, crude natural product mixtures are tested for a specific pharmacological activity and then active ingredient is purified. The retrospective identification of the molecular targets that underlie the observed phenotypic responses is called target deconvolution. Unveiling the cellular targets of a given molecular entity is necessary for a better understanding of its mechanism of action, prediction of potential pharmacological activities as well as plausible side effects and off-target toxicities.

Affinity-based target deconvolution method is complicated by the risk of identifying interactions with proteins that have no pharmacological relevance (false positives), despite being targets of the compound. Therefore, activity- or phenotype-based assays are essential to discriminate between positive and false-positive interactions and to confirm true functional effects [19]. Another major challenge in affinity chromatography-coupled MS technology is the non-specific interaction of proteins with the immobilized support and/or linker [24]. In the current investigation, the referred problem was minimized by applying a control column and eliminating the cellular proteins that are more prone to bind the solid support.

Proteomic findings revealed that crocin binds to a wide range of cellular proteins such as structural proteins, membrane transporters, and enzymes involved in ATP and redox homeostasis and signal transduction. Beta-actin-like protein 2 was identified as one of the target proteins of crocin. Actin filaments help maintaining cell morphology and mediate functions such as adhesion, motility, exocytosis, endocytosis and cell division. Natural products like cytochalasin and jasklapinolide that interact with actin polymerization have cytotoxic effects [25].

Tubulin beta 3 and 6 are also cytoskeletal proteins that interact with crocin. Microtubules are long, hollow, cylindrical protein polymers composed of α/β-tubulin heterodimers. An important function of microtubules is to move cellular structures such as chromosomes, mitotic spindles and other organelles inside the cell [26]. Several microtubule-inhibiting agents such as vincristine, vinblastine, taxol and colchicine have shown potent activity against the proliferation of various cancer cells [27]. Crocin has been reported to significantly inhibit the growth of different types of cancerous cell lines such as colorectal cancer cells [11]. Effects of crocin on tubulin polymerization has been already studied [28]. Crocin may alter the tubulin polymerization through direct binding.

ATP synthase is a key enzyme of mitochondrial energy conversion [29]. Ahmad and Laughlin [30] discussed that dietary polyphenols and amphibian antimicrobial/antitumor peptides inhibit ATP synthase. Inhibition of ATP synthase may cause energy deprivation and increase ROS production. High ROS content induces cellular necrosis and/or apoptosis [29]. Our experiment showed that crocin may physically interact with this enzyme, which is consistent with the findings of a previous study with safranal as another important constituent of saffron [31]. However, contrasting evidence has shown that crocin reduces ROS generation in cells exposed to acry1amide [32]. Overall, the majority of previous findings favor the antioxidant role of crocin and this may be due to the inhibitory effect of this phytochemical on other sources of ROS production, in particular lipid peroxidation, as well enhancement of free radical neutralization via stimulating the activity of superoxide dismutase and increasing intracellular glutathione content [33,34].

Creatine kinase (CK) catalyzes transfer of phosphate group from ATP to creatine to produce phosphocreatine and *vice versa*. CK works as an energy buffer and is found in tissues with high and/or fluctuating energy demand such as heart, muscle and brain [35]. Incubation of crocin-resin with brain homogenate showed that crocin has affinity for CK-B. Any change in CK activity may affect energy homeostasis in cell. Dahlstedt and Westerblad [36] showed that creatine kinase inhibition may reduce the rate of fatigue induced by decrease in tetanic Ca^2+^ in mouse skeletal muscle [37]. The oral administration of crocetin (another carotenoid of saffron) has been reported to improve physical capacity during fatigue-induced workload tests in men [38].

Crocin was also found to interact with cytochrome c1 and cytochrome b-c1. The most conserved role of these cytochromes is in the electron transport chain and oxidative phosphorylation. Moreover, cytochrome c release into the cytosol is particularly associated with activation of the intrinsic apoptotic pathway [39]. In previous studies, saffron carotenoids including crocin have been shown to modulate apoptosis through different mechanisms such as inhibition of ROS production [32,33] and direct interaction with caspase-3 and caspase-8 [14,15]. Crocin has been reported to promote apoptosis in tumor cells while exerting anti-apoptotic effects in non-tumor cells [40,41]. In view of the present finding, interaction of crocin with cytochrome c might play an important role in the stimulatory and inhibitory activities of this phytochemical on the apoptosis pathway and deserves further attention.

V-ATPase inhibitors such as bafilomycin A1 may induce apoptosis through intracellular acidosis. Effects of physical binding of crocin on V-ATPase activity should be studied in detail. It has been discussed that V-ATPase inhibitors can potentially be used in the treatment of solid tumors with overexpressed levels of this enzyme [42].

Crocin may also interact with biosynthetic pathways through direct interaction with acetyl-coenzyme A acetyl transferases (ACAT). ACAT converts two units of acetyl-CoA to acetoacetyl CoA in poly beta-hydroxybutyrate synthesis or steroid biogenesis [43].

Our study also showed that crocin binds to proteasome α type 4 and 6. Proteasome degrades misfolded and/or ubiquitin-tagged proteins. Proteasome inhibitors, like disulfiram, have been recently studied for cancer therapy [44,45]. Affinity of crocin for proteasome may explain its cytotoxic effect at higher concentrations.

Another crocin target was identified as 14-3-3 protein beta/alpha. 14-3-3 proteins are implicated in the regulation of key proteins such as Raf, bad, and Cbl, and are implicated in various biological processes such as signal transduction, transcriptional control, cell proliferation, apoptosis and ion channel physiology [46]. 14-3-3 protein zeta interacts with insulin resistance substrate-1 (IRS-1) protein and might therefore play a role in regulating insulin sensitivity. Crocin and safranal have been reported to reduce blood glucose and HbA1c levels but increase blood insulin levels significantly without any significant effect on liver and kidney functions in alloxan-induced diabetic rats [47].

Affinity of crocin for heat shock protein 60 may explain some the protective effects of saffron. Heat shock proteins are chaperones that assist proteins for proper folding, stability and transport across cellular membranes [48]. There is evidence indicating the cardioprotective effects of saffron and improvement of histopathologic and biochemical parameters in the cardiac tissue following stress [49,50]. Overexpression of heat shock protein 60 in myocardium is a defensive biological mechanism for the preservation of cardiac function upon exposure to cardiotoxic agents or other stressors [51]. Physical interaction of crocin with heat shock protein 60 might influence the function of this chaperone and improves its protective effects.

Peroxiredoxin-2 also shows some degrees of physical affinity to crocin. Peroxiredoxin-2 reduces the level of H_2_O_2_ in cells. Both crocin and peroxiredoxin may play an antioxidant protective role in cells. Physical interaction of these two enzymes may alter their antioxidant capacity.

In summary, the present data revealed that tubulin beta-3 chain, tubulin beta-6 chain, ATP synthase subunit beta, beta-actin-like protein 2, 14-3-3 protein beta/alpha, V-type proton ATPase, 60 kDa heat shock protein, creatine kinase B-type, peroxiredoxin-2, cytochrome b-c1 complex, cytochrome c1, heme protein, acetyl-CoA acetyltransferase, proteasome subunit alpha type-4 and type-6, protein disulfide-isomerase and delta-aminolevulinic acid dehydratase could serve as potential cellular targets for crocin. Although physical interaction of crocin with these proteins may explain some of its pharmacological effects, activity- or phenotype-based assays are essential to discriminate between positive and false-positive interactions.

## Abbreviations

PMSF: Phenylmethylsulfonyl fluoride; DADPA: Diaminodipropylamine; CHAPS: (3-[(3-Cholamidopropyl)-dimethylammonio]-1-propane sulfonate); DTT: Dithiothreitol; IPGs: Immobilized pH gradient; SDS-PAGE: Sodium dodecyl sulfate-polyacrylamide gel electrophoresis; IEF: Isoelectric focusing; MALDI: Matrix-assisted laser desorption ionization; TOF: Time-of-flight; CoA: Coenzyme A; EDTA: Ethylenediaminetetraacetic acid; EGTA: Ethylene glycol tetraacetic acid; IRS-1: Insulin resistance substrate-1; ATP: Adenosine triphosphate; ACAT: Acetyl-coenzyme A acetyl transferases; CK: Creatine kinase; MS: Mass spectrometry.

## Competing interests

The authors have no competing interest to declare.

## Authors’ contributions

KA, HH and MR conceived the study and designed the experiments. SM and AH did the experimental works. KA, SM, AH and AS performed literature review and were involved in the drafting and submission of the manuscript. All authors read and approved the final manuscript.

## Supplementary Material

Additional file 1 Results of MASCOT search are available as supporting information. Click here for file
