# Supplementary material for: Proteomic screening of molecular targets of crocin
Source: Daru. 2014 Jan 6;22(1):5. doi: 10.1186/2008-2231-22-5 (PMC3922837; doi:10.1186/2008-2231-22-5)
Supplement: Additional file 1 — Results of MASCOT search are available as supporting information. [file 2008-2231-22-5-S1.pdf]

Results from MASCOT search

1. Beta-actin-like protein 2

Analysis Information

Report Type	Protein-Peptide Summary by Spot	Analysis Type	Combined (MS+MS/MS)
Sample Set Name	2011-05-04 [001300010097-2011-03-18-1]	Database	SwissProt
Analysis Name	Khalil-Medicinal Chemistry[Mus Swissprot]	Creation Date	05/04/2011 13:12:29
Reported By	05/04/2011 17:22:24 - admin	Last Modified	05/04/2011 13:18:54

MS Acq. : Proc. Methods	(Unspecified) : (Unspecified)
Interpretation Method	(Unspecified)

Gel Idx/Pos	324/N2	Instr./Gel Origin	Ak115/2011-03-18-1	Process Status	Analysis Succeeded
Plate [#] Name	[1] 001300010097	Instrument Sample Name		Spectra	6

Rank	Protein Name	Accession No.	Protein Score	Protein Score C. I. %	Total Ion Score	Total Ion C. I. %	Protein MW	Protein PI	Spot Name	Pep. Count	User name	Department
1	RecName: Full=Beta-actin-like protein 2; AltName: Full=Kappa-actin	gi|81895966|sp|Q8BFZ3.1|ACTBL_MOUSE	215	100	175	100	42319.1	5.3	A-H-2	7	Khalil	Medicinal Chemistry

Peptide Information

Calc. Mass	Obsrv. Mass	± da	± ppm	Start Seq.	End Seq.	Sequence	Ion Score	C. I. %	Modification	Rank	Result Type
923.5672	923.6219	0.0547	59	330	337	IIAPPERK					Mascot
1036.6514	1036.7253	0.0739	71	328	336	IKIIAPPER					Mascot
1171.571	1171.6542	0.0832	71	41	51	HQGVMVGMGQK					Mascot
1623.8411	1623.9576	0.1165	72	179	192	LDLAGRDLTDYLMK					Mascot
1639.8359	1639.9587	0.1228	75	179	192	LDLAGRDLTDYLMK			Oxidation (M)[13]		Mascot
1790.892	1791.0049	0.1129	63	240	255	SYELPDGQVITIGNER	114	100			Mascot
1790.892	1791.0049	0.1129	63	240	255	SYELPDGQVITIGNER					Mascot
1954.0645	1954.1871	0.1226	63	97	114	VAPDEHPILLTEAPLNPK	61	99.998			Mascot
1954.0645	1954.1871	0.1226	63	97	114	VAPDEHPILLTEAPLNPK					Mascot
3183.6143	3183.8113	0.197	62	149	178	TTGIVMDSGDGVTHTVPI [illegible]					Mascot

2. Cytochrome b-c1 complex subunit 1

Analysis Information

Report Type	Protein-Peptide Summary by Spot	Analysis Type	Combined (MS+MS/MS)
Sample Set Name	2011-05-04 [001300010097-2011-03-18-1]	Database	SwissProt
Analysis Name	Khalil-Medicinal Chemistry[Mus Swissprot]	Creation Date	05/04/2011 13:12:29
Reported By	05/04/2011 17:22:25 - admin	Last Modified	05/04/2011 13:18:54

MS Acq. : Proc. Methods	(Unspecified) : (Unspecified)
Interpretation Method	(Unspecified)

Gel Idx/Pos	327/N5	Instr./Gel Origin	Ak115/2011-03-18-1	Process Status	Analysis Succeeded
Plate [#] Name	[1] 001300010097	Instrument Sample Name		Spectra	6

Rank	Protein Name	Accession No.	Protein Score	Protein Score C. I. %	Total Ion Score	Total Ion C. I. %	Protein MW	Protein PI	Spot Name	Pep. Count	User name	Department
1	RecName: Full=Cytochrome b-c1 complex subunit 1, mitochondrial; AltName: Full=Complex III subunit 1	gi|14548301|sp|Q9CZ13.1|QCR1_MOUSE	265	100	196	100	53419.7	5.75	A-L-1	11	Khalil	Medicinal Chemistry

Peptide Information

Calc. Mass	Obsrv. Mass	± da	± ppm	Start Seq.	End Seq.	Sequence	Ion Score	C. I. %	Modification	Rank	Result Type
912.4396	912.4569	0.0173	19	473	479	SGMFWLR			Oxidation (M)[3]		Mascot
1059.5081	1059.5363	0.0282	27	473	480	SGMFWLRF			Oxidation (M)[3]		Mascot
1100.5735	1100.6115	0.038	35	424	432	IPLAEWESR					Mascot
1110.5426	1110.5802	0.0376	34	214	222	TDLTDYLNR					Mascot
1202.6198	1202.6586	0.0388	32	433	442	IQEVDAQMLR					Mascot
1218.6147	1218.6503	0.0356	29	433	442	IQEVDAQMLR			Oxidation (M)[8]		Mascot
1256.6746	1256.7067	0.0321	26	423	432	RIPLAEWESR	41	99.719			Mascot
1256.6746	1256.7067	0.0321	26	423	432	RIPLAEWESR					Mascot
1538.7479	1538.8057	0.0578	38	379	392	LCTSATESEVTRGK			Carbamidomethyl (C)[2]		Mascot
1605.7577	1605.7983	0.0406	25	256	269	VYEEDAVPGLTPCR	71	100	Carbamidomethyl (C)[13]		Mascot
1605.7577	1605.7983	0.0406	25	256	269	VYEEDAVPGLTPCR			Carbamidomethyl (C)[13]		Mascot
1646.8132	1646.8577	0.0445	27	112	126	EVESIGAHLNAYSTR					Mascot
2053.9971	2054.0491	0.052	25	397	415	NALVSHLDGTTPVCEDIGR	84	100	Carbamidomethyl (C)[14]		Mascot
2053.9971	2054.0491	0.052	25	397	415	NALVSHLDGTTPVCEDIGR			Carbamidomethyl (C)[14]		Mascot
2602.3259	2602.3831	0.0572	22	112	134	EVESIGAHLNAYSTREHTAYLIK					Mascot

3. ATP synthase subunit beta

Analysis Information

Report Type	Protein-Peptide Summary by Spot	Analysis Type	Combined (MS+MS/MS)
Sample Set Name	2011-05-04 [001300010097-2011-03-18-1]	Database	SwissProt
Analysis Name	Khalil-Medicinal Chemistry[Mus Swissprot]	Creation Date	05/04/2011 13:12:29
Reported By	05/04/2011 17:22:26 - admin	Last Modified	05/04/2011 13:18:54

MS Acq. : Proc. Methods	(Unspecified) : (Unspecified)
Interpretation Method	(Unspecified)

Gel Idx/Pos	328/N6	Instr./Gel Origin	Ak115/2011-03-18-1	Process Status	Analysis Succeeded
Plate [#] Name	[1] 001300010097	Instrument Sample Name		Spectra	6

Rank	Protein Name	Accession No.	Protein Score	Protein Score C. I. %	Total Ion Score	Total Ion C. I. %	Protein MW	Protein PI	Spot Name	Pep. Count	User name	Department
1	RecName: Full=ATP synthase subunit beta, mitochondrial; Flags: Precursor	gi|20455479|sp|P56480.2|ATPB_MOUSE	485	100	367	100	56265.5	5.19	A-L-2	15	Khalil	Medicinal Chemistry

Peptide Information

Calc. Mass	Obsrv. Mass	± da	± ppm	Start Seq.	End Seq.	Sequence	Ion Score	C. I. %	Modification	Rank	Result Type
1262.6409	1262.7026	0.0617	49	110	121	TIAMDGTEGLVR					Mascot
1385.7094	1385.7786	0.0692	50	144	155	IMNVIGEPIDER					Mascot
1401.7042	1401.7753	0.0711	51	144	155	IMNVIGEPIDER			Oxidation (M)[2]		Mascot
1406.681	1406.7507	0.0697	50	226	239	AHGGYSVFAGVGER	127	100			Mascot
1406.681	1406.7507	0.0697	50	226	239	AHGGYSVFAGVGER					Mascot
1435.7539	1435.8242	0.0703	49	311	324	FTQAGSEVSALLGR					Mascot
1439.7893	1439.8893	0.1	69	282	294	VALTGLTVAEYFR					Mascot
1601.8104	1601.886	0.0756	47	265	279	VALVYGQMNEPPGAR	55	99.992			Mascot
1601.8104	1601.886	0.0756	47	265	279	VALVYGQMNEPPGAR					Mascot
1617.8053	1617.8818	0.0765	47	265	279	VALVYGQMNEPPGAR			Oxidation (M)[8]		Mascot
1650.9174	1650.9941	0.0767	46	95	109	LVLEVAQHLGESTVR	78	100			Mascot
1650.9174	1650.9941	0.0767	46	95	109	LVLEVAQHLGESTVR					Mascot
1780.9625	1781.047	0.0845	47	144	159	IMNVIGEPIDERGPIK					Mascot
1796.9575	1797.0184	0.0609	34	144	159	IMNVIGEPIDERGPIK			Oxidation (M)[2]		Mascot
1842.8802	1842.9714	0.0912	49	407	422	IMDPNIVGNEHYDVAR					Mascot
1858.8752	1858.9777	0.1025	55	407	422	IMDPNIVGNEHYDVAR			Oxidation (M)[2]		Mascot
1919.0959	1919.1879	0.092	48	125	143	VLDSGAPIKIPVGPETLG	107	100			Mascot

4. Tubulin beta-3 chain

Analysis Information

Report Type	Protein-Peptide Summary by Spot	Analysis Type	Combined (MS+MS/MS)
Sample Set Name	2011-05-04 [001300010097-2011-03-18-1]	Database	SwissProt
Analysis Name	Khalil-Medicinal Chemistry[Mus Swissprot]	Creation Date	05/04/2011 13:12:29
Reported By	05/04/2011 17:22:27 - admin	Last Modified	05/04/2011 13:18:54

MS Acq. : Proc. Methods	(Unspecified) : (Unspecified)
Interpretation Method	(Unspecified)

Gel Idx/Pos	330/N8	Instr./Gel Origin	Ak115/2011-03-18-1	Process Status	Analysis Succeeded
Plate [#] Name	[1] 001300010097	Instrument Sample Name		Spectra	6

Rank	Protein Name	Accession No.	Protein Score	Protein Score C. I. %	Total Ion Score	Total Ion C. I. %	Protein MW	Protein PI	Spot Name	Pep. Count	User name	Department
1	RecName: Full=Tubulin beta-3 chain	gi|20455323|sp|Q9ERD7.1|TBB3_MOUSE	258	100	185	100	50842.4	4.82	H-B-1	11	Khalil	Medicinal Chemistry

Peptide Information

Calc. Mass	Obsrv. Mass	± da	± ppm	Start Seq.	End Seq.	Sequence	Ion Score	C. I. %	Modification	Rank	Result Type
1069.6041	1069.5824	-0.0217	-20	310	318	YLTVATVFR					Mascot
1130.5953	1130.5734	-0.0219	-19	242	251	FPGQLNADLR	72	100			Mascot
1130.5953	1130.5734	-0.0219	-19	242	251	FPGQLNADLR					Mascot
1143.6343	1143.6111	-0.0232	-20	253	262	LAVNMVPFPR	46	99.865			Mascot
1143.6343	1143.6111	-0.0232	-20	253	262	LAVNMVPFPR					Mascot
1159.6293	1159.6006	-0.0287	-25	253	262	LAVNMVPFPR	11	0	Oxidation (M)[5]		Mascot
1159.6293	1159.6006	-0.0287	-25	253	262	LAVNMVPFPR			Oxidation (M)[5]		Mascot
1229.5983	1229.576	-0.0223	-18	381	390	ISEQFTAMFR					Mascot
1245.5933	1245.5662	-0.0271	-22	381	390	ISEQFTAMFR			Oxidation (M)[8]		Mascot
1258.6902	1258.6639	-0.0263	-21	242	252	FPGQLNADLRK					Mascot
1385.6995	1385.6698	-0.0297	-21	381	391	ISEQFTAMFRR					Mascot
1390.6882	1390.6528	-0.0354	-25	325	336	EVDEQMLAIQSK					Mascot
1406.6832	1406.6526	-0.0306	-22	325	336	EVDEQMLAIQSK			Oxidation (M)[6]		Mascot
1604.8406	1604.7898	-0.0508	-32	263	276	LHFFMPGFAPLTAR					Mascot
1615.8359	1615.7975	-0.0384	-24	63	77	AILVDLEPGTMDSVR	67	100			Mascot
1615.8359	1615.7975	-0.0384	-24	63	77	AILVDLEPGTMDSVR					Mascot
1620.8356	1620.7953	-0.0403	-25	263	276	LHFFMPGFAPLTAR			Oxidation (M)[5]		Mascot

5. Tubulin beta-6 chain

Analysis Information

Report Type	Protein-Peptide Summary by Spot	Analysis Type	Combined (MS+MS/MS)
Sample Set Name	2011-05-04 [001300010097-2011-03-18-1]	Database	SwissProt
Analysis Name	Khalil-Medicinal Chemistry[Mus Swissprot]	Creation Date	05/04/2011 13:12:29
Reported By	05/04/2011 17:22:27 - admin	Last Modified	05/04/2011 13:18:54

MS Acq. : Proc. Methods	(Unspecified) : (Unspecified)
Interpretation Method	(Unspecified)

Gel Idx/Pos	330/N8	Instr./Gel Origin	Ak115/2011-03-18-1	Process Status	Analysis Succeeded
Plate [#] Name	[1] 001300010097	Instrument Sample Name		Spectra	6

Rank	Protein Name	Accession No.	Protein Score	Protein Score C. I. %	Total Ion Score	Total Ion C. I. %	Protein MW	Protein PI	Spot Name	Pep. Count	User name	Department
	RecName: Full=Tubulin beta-6 chain	gi|68775966|sp|Q922F4.1|TBB6_MOUSE	169	100	118	100	50514.3	4.8	H-B-1	9	Khalil	Medicinal Chemistry

Peptide Information

Calc. Mass	Obsrv. Mass	± da	± ppm	Start Seq.	End Seq.	Sequence	Ion Score	C. I. %	Modification	Rank	Result Type
1069.6041	1069.5824	-0.0217	-20	310	318	YLTVATVFR					Mascot
1077.5323	1077.5089	-0.0234	-22	155	162	IREEYPDR					Mascot
1130.5953	1130.5734	-0.0219	-19	242	251	FPGQLNADLR	72	100			Mascot
1130.5953	1130.5734	-0.0219	-19	242	251	FPGQLNADLR					Mascot
1143.6343	1143.6111	-0.0232	-20	253	262	LAVNMVPFPR	46	99.865			Mascot
1143.6343	1143.6111	-0.0232	-20	253	262	LAVNMVPFPR					Mascot
1159.6293	1159.6006	-0.0287	-25	253	262	LAVNMVPFPR	11	0	Oxidation (M)[5]		Mascot
1159.6293	1159.6006	-0.0287	-25	253	262	LAVNMVPFPR			Oxidation (M)[5]		Mascot
1258.6902	1258.6639	-0.0263	-21	242	252	FPGQLNADLRK					Mascot
1604.8406	1604.7898	-0.0508	-32	263	276	LHFFMPGFAPLTAR					Mascot
1620.8356	1620.7953	-0.0403	-25	263	276	LHFFMPGFAPLTAR			Oxidation (M)[5]		Mascot
1696.8329	1696.7893	-0.0436	-26	337	350	NSSYFVEWIPNNVK					Mascot
1700.8862	1700.8041	-0.0821	-48	310	324	YLTVATVFRGPMSMK					Mascot
1958.9818	1958.9427	-0.0391	-20	104	121	GHYTEGAELVDSVLDVV[illegible]					Mascot

6. 14-3-3 protein beta/alpha

Analysis Information

Report Type	Protein-Peptide Summary by Spot	Analysis Type	Combined (MS+MS/MS)
Sample Set Name	2011-05-04 [001300010097-2011-03-18-1]	Database	SwissProt
Analysis Name	Khalil-Medicinal Chemistry[Mus Swissprot]	Creation Date	05/04/2011 13:12:29
Reported By	05/04/2011 17:22:28 - admin	Last Modified	05/04/2011 13:18:54

MS Acq. : Proc. Methods	(Unspecified) : (Unspecified)
Interpretation Method	(Unspecified)

Gel Idx/Pos	332/N10	Instr./Gel Origin	Ak115/2011-03-18-1	Process Status	Analysis Succeeded
Plate [#] Name	[1] 001300010097	Instrument Sample Name		Spectra	6

Rank	Protein Name	Accession No.	Protein Score	Protein Score C. I. %	Total Ion Score	Total Ion C. I. %	Protein MW	Protein PI	Spot Name	Pep. Count	User name	Department
1	RecName: Full=14-3-3 protein beta/alpha; AltName: Full=Protein kinase C inhibitor protein 1; Short=	gi|18202836|sp|Q9CQV8.3|1433B_MOUSE	160	100	104	100	28182.9	4.77	H-B-3	9	Khalil	Medicinal Chemistry

Peptide Information

Calc. Mass	Obsrv. Mass	± da	± ppm	Start Seq.	End Seq.	Sequence	Ion Score	C. I. %	Modification	Rank	Result Type
903.5145	903.4909	-0.0236	-26	63	70	VISSIEQK					Mascot
907.5247	907.4935	-0.0312	-34	44	51	NLLSVAYK					Mascot
916.4345	916.4106	-0.0239	-26	123	129	MKGDYFR					Mascot
932.4294	932.4056	-0.0238	-26	123	129	MKGDYFR			Oxidation (M)[1]		Mascot
1108.5569	1108.5365	-0.0204	-18	161	169	EMQPTHPIR					Mascot
1124.5518	1124.5269	-0.0249	-22	161	169	EMQPTHPIR			Oxidation (M)[2]		Mascot
1189.6609	1189.6326	-0.0283	-24	215	224	DSTLIMQLLR					Mascot
1205.6559	1205.6271	-0.0288	-24	215	224	DSTLIMQLLR			Oxidation (M)[6]		Mascot
1236.6517	1236.6355	-0.0162	-13	160	169	KEMQPTHPIR					Mascot
1503.8641	1503.8271	-0.037	-25	44	57	NLLSVAYKNVVGAR					Mascot
1598.7405	1598.7085	-0.032	-20	30	43	AVTEQGHELSNEER					Mascot
1812.8254	1812.7815	-0.0439	-24	14	29	LAEQAERYDDMAAAMK	104	100			Mascot
1812.8254	1812.7815	-0.0439	-24	14	29	LAEQAERYDDMAAAMK					Mascot
1828.8204	1828.772	-0.0484	-26	14	29	LAEQAERYDDMAAAMK	29	96.284	Oxidation (M)[15]		Mascot
1828.8204	1828.772	-0.0484	-26	14	29	LAEQAERYDDMAAAMK			Oxidation (M)[11]		Mascot
1844.8153	1844.7729	-0.0424	-23	14	29	LAEQAERYDDMAAAMK			Oxidation (M)[11,15]		Mascot

7. V-type proton ATPase catalytic subunitA

Analysis Information

Report Type	Protein-Peptide Summary by Spot	Analysis Type	Combined (MS+MS/MS)
Sample Set Name	2011-05-04 [001300010097-2011-03-18-1]	Database	SwissProt
Analysis Name	Khalil-Medicinal Chemistry[Mus Swissprot]	Creation Date	05/04/2011 13:12:29
Reported By	05/04/2011 17:22:28 - admin	Last Modified	05/04/2011 13:18:54

MS Acq. : Proc. Methods	(Unspecified) : (Unspecified)
Interpretation Method	(Unspecified)

Gel Idx/Pos	333/N11	Instr./Gel Origin	Ak115/2011-03-18-1	Process Status	Analysis Succeeded
Plate [#] Name	[1] 001300010097	Instrument Sample Name		Spectra	6

Rank	Protein Name	Accession No.	Protein Score	Protein Score C. I. %	Total Ion Score	Total Ion C. I. %	Protein MW	Protein PI	Spot Name	Pep. Count	User name	Department
1	RecName: Full=V-type proton ATPase catalytic subunit A; Short=V-ATPase subunit A; AltName: Full=V-A	gi|145559539|sp|P50516.2|VATA_MOUSE	113	100	79	100	68624.7	5.42	H-B-4	8	Khalil	Medicinal Chemistry

Peptide Information

Calc. Mass	Obsrv. Mass	± da	± ppm	Start Seq.	End Seq.	Sequence	Ion Score	C. I. %	Modification	Rank	Result Type
902.5053	902.4855	-0.0198	-22	121	129	GVNVSALSR					Mascot
1248.6558	1248.6229	-0.0329	-26	203	212	FSMVQVWPVR					Mascot
1262.678	1262.636	-0.042	-33	130	139	DIKWEFIPSK					Mascot
1264.6508	1264.6208	-0.03	-24	203	212	FSMVQVWPVR			Oxidation (M)[3]		Mascot
1308.7271	1308.6749	-0.0522	-40	45	56	VGHSELVGEIIR					Mascot
1316.7434	1316.7129	-0.0305	-23	221	232	LPANHPLLTGQR	79	100			Mascot
1316.7434	1316.7129	-0.0305	-23	221	232	LPANHPLLTGQR					Mascot
1319.655	1319.647	-0.008	-6	553	564	RAVETTAQSDNK					Mascot
1515.7948	1515.756	-0.0388	-26	309	323	TALVANTSNMPVAAR					Mascot
1531.7897	1531.7384	-0.0513	-33	309	323	TALVANTSNMPVAAR			Oxidation (M)[10]		Mascot
1781.8527	1781.7861	-0.0666	-37	365	381	LAEMPADSGYPAYLGAR					Mascot
1797.8477	1797.7947	-0.053	-29	365	381	LAEMPADSGYPAYLGAR			Oxidation (M)[4]		Mascot

8. 60 kDa heat shock protein

Analysis Information

Report Type	Protein-Peptide Summary by Spot	Analysis Type	Combined (MS+MS/MS)
Sample Set Name	2011-05-04 [001300010097-2011-03-18-1]	Database	SwissProt
Analysis Name	Khalil-Medicinal Chemistry[Mus Swissprot]	Creation Date	05/04/2011 13:12:29
Reported By	05/04/2011 17:22:28 - admin	Last Modified	05/04/2011 13:18:54

MS Acq. : Proc. Methods	(Unspecified) : (Unspecified)
Interpretation Method	(Unspecified)

Gel Idx/Pos	333/N11	Instr./Gel Origin	Ak115/2011-03-18-1	Process Status	Analysis Succeeded
Plate [#] Name	[1] 001300010097	Instrument Sample Name		Spectra	6

Rank	Protein Name	Accession No.	Protein Score	Protein Score C. I. %	Total Ion Score	Total Ion C. I. %	Protein MW	Protein PI	Spot Name	Pep. Count	User name	Department
	heat shock protein hsp60, hsp60=chaperonin [mice, Peptide, 573 aa]	gi|247242	348	100	274	100	61111.5	6.35	H-B-4	14	Khalil	Medicinal Chemistry

Peptide Information

Calc. Mass	Obsrv. Mass	± da	± ppm	Start Seq.	End Seq.	Sequence	Ion Score	C. I. %	Modification	Rank	Result Type
901.5353	901.5007	-0.0346	-38	397	405	LSDGVAVLK					Mascot
912.5876	912.5194	-0.0682	-75	293	301	VGLQVVAVK					Mascot
941.6141	941.5929	-0.0212	-23	463	470	IGIEIIKR					Mascot
960.5109	960.4918	-0.0191	-20	421	429	VTDALNATR					Mascot
961.4849	961.4778	-0.0071	-7	302	310	APGFGDNRK					Mascot
1008.5359	1008.514	-0.0219	-22	122	130	SIAKEGFEK					Mascot
1017.5938	1017.539	-0.0548	-54	73	82	VTKDGVTVAK					Mascot
1097.6677	1097.6472	-0.0205	-19	517	526	GIIDPTKVVR					Mascot
1215.658	1215.6248	-0.0332	-27	482	493	NAGVEGSLIVEK					Mascot
1344.7158	1344.6816	-0.0342	-25	61	72	TVIIEQSWGSPK					Mascot
1389.705	1389.6661	-0.0389	-28	222	233	GYISPYFINTSK	38	96.123			Mascot
1389.705	1389.6661	-0.0389	-28	222	233	GYISPYFINTSK					Mascot
1630.9084	1630.8596	-0.0488	-30	345	359	VGEVIVTKDDAMLLK					Mascot
1905.0552	1905.0034	-0.0518	-27	251	268	ISSVQSIVPALEIANAHR					Mascot
2560.2485	2560.1692	-0.0793	-31	97	121	LVQDVANNTNEEAGDGT TTATVLAR	236	100			Mascot
2560.2485	2560.1692	-0.0793	-31	97	121	LVQDVANNTNEEAGDGT [illegible]					Mascot

9. Creatine kinase B-type

Analysis Information

Report Type	Protein-Peptide Summary by Spot	Analysis Type	Combined (MS+MS/MS)
Sample Set Name	2011-05-04 [001300010097-2011-03-18-1]	Database	SwissProt
Analysis Name	Khalil-Medicinal Chemistry[Mus Swissprot]	Creation Date	05/04/2011 13:12:29
Reported By	05/04/2011 17:22:29 - admin	Last Modified	05/04/2011 13:18:54

MS Acq. : Proc. Methods	(Unspecified) : (Unspecified)
Interpretation Method	(Unspecified)

Gel Idx/Pos	334/N12	Instr./Gel Origin	Ak115/2011-03-18-1	Process Status	Analysis Succeeded
Plate [#] Name	[1] 001300010097	Instrument Sample Name		Spectra	6

Rank	Protein Name	Accession No.	Protein Score	Protein Score C. I. %	Total Ion Score	Total Ion C. I. %	Protein MW	Protein PI	Spot Name	Pep. Count	User name	Department
1	RecName: Full=Creatine kinase B-type; AltName: Full=B-CK; AltName: Full=Creatine kinase B chain	gi|417208|sp|Q04447.1|KCRB_MOUSE	388	100	295	100	42971.4	5.4	H-B-5	12	Khalil	Medicinal Chemistry

Peptide Information

Calc. Mass	Obsrv. Mass	± da	± ppm	Start Seq.	End Seq.	Sequence	Ion Score	C. I. %	Modification	Rank	Result Type
1031.5554	1031.5242	-0.0312	-30	359	366	LLIEMEQR					Mascot
1047.5503	1047.5157	-0.0346	-33	359	366	LLIEMEQR			Oxidation (M)[5]		Mascot
1154.5623	1154.5215	-0.0408	-35	243	252	GGNMKEVFTR			Oxidation (M)[4]		Mascot
1159.5491	1159.5503	0.0012	1	2	11	PFSNSHNTQK					Mascot
1246.6313	1246.5916	-0.0397	-32	87	96	DLFDPIIEER	69	100			Mascot
1246.6313	1246.5916	-0.0397	-32	87	96	DLFDPIIEER					Mascot
1303.7256	1303.6785	-0.0471	-36	33	43	VLTPELYAELR					Mascot
1502.8577	1502.7997	-0.058	-39	33	45	VLTPELYAELRAK					Mascot
1602.8334	1602.7755	-0.0579	-36	157	172	LAVEALSSLDGDLSGR	97	100			Mascot
1602.8334	1602.7755	-0.0579	-36	157	172	LAVEALSSLDGDLSGR					Mascot
1672.821	1672.7568	-0.0642	-38	367	381	LEQGQAIDDLMPAQK			Oxidation (M)[11]		Mascot
1964.9308	1964.8582	-0.0726	-37	321	341	GTGGVDTAAVGGVFDVSNADR	129	100			Mascot
1964.9308	1964.8582	-0.0726	-37	321	341	GTGGVDTAAVGGVFDVSNADR					Mascot
2121.032	2120.9487	-0.0833	-39	320	341	RGTGGVDTAAVGGVFDVSNADR					Mascot
2201.9556	2201.8713	-0.0843	-38	14	32	FPAEDEFPDLSSHNNHMAK			Oxidation (M)[17]		Mascot
2701.3535	2701.2354	-0.1181	-44	359	381	LLIEMEQRLEQGQAIDDLMPAQK			Oxidation (M)[5,19]		Mascot

10. Peroxiredoxin-2

Analysis Information

Report Type	Protein-Peptide Summary by Spot	Analysis Type	Combined (MS+MS/MS)
Sample Set Name	2011-05-04 [001300010097-2011-03-18-1]	Database	SwissProt
Analysis Name	Khalil-Medicinal Chemistry[Mus Swissprot]	Creation Date	05/04/2011 13:12:29
Reported By	05/04/2011 17:22:30 - admin	Last Modified	05/04/2011 13:18:54

MS Acq. : Proc. Methods	(Unspecified) : (Unspecified)
Interpretation Method	(Unspecified)

Gel Idx/Pos	335/N13	Instr./Gel Origin	Ak115/2011-03-18-1	Process Status	Analysis Succeeded
Plate [#] Name	[1] 001300010097	Instrument Sample Name		Spectra	6

Rank	Protein Name	Accession No.	Protein Score	Protein Score C. I. %	Total Ion Score	Total Ion C. I. %	Protein MW	Protein PI	Spot Name	Pep. Count	User name	Department
1	RecName: Full=Peroxiredoxin-2; AltName: Full=Thiol-specific antioxidant protein; Short=TSA; AltName	gi|2499469|sp|Q61171.3|PRDX2_MOUSE	283	100	240	100	21936.1	5.2	H-B-6	6	Khalil	Medicinal Chemistry

Peptide Information

Calc. Mass	Obsrv. Mass	± da	± ppm	Start Seq.	End Seq.	Sequence	Ion Score	C. I. %	Modification	Rank	Result Type
937.4373	937.4006	-0.0367	-39	120	127	NDEGIAYR					Mascot
976.488	976.4149	-0.0731	-75	1	10	MASGNAQIGK					Mascot
1023.5469	1023.4988	-0.0481	-47	27	34	EIKLSDYR					Mascot
1108.5997	1108.5618	-0.0379	-34	110	119	SLSQNYGVLK					Mascot
1211.6743	1211.631	-0.0433	-36	140	150	QITVNDLPVGR	91	100			Mascot
1211.6743	1211.631	-0.0433	-36	140	150	QITVNDLPVGR					Mascot
2027.0192	2026.9398	-0.0794	-39	110	127	SLSQNYGVLKNDEGIAYR	149	100			Mascot
2027.0192	2026.9398	-0.0794	-39	110	127	SLSQNYGVLKNDEGIAYR					Mascot

11. Cytochrome b-c1 complex subunit 2

Analysis Information

Report Type	Protein-Peptide Summary by Spot	Analysis Type	Combined (MS+MS/MS)
Sample Set Name	2011-05-04 [001300010097-2011-03-18-1]	Database	SwissProt
Analysis Name	Khalil-Medicinal Chemistry[Mus Swissprot]	Creation Date	05/04/2011 13:12:29
Reported By	05/04/2011 17:22:32 - admin	Last Modified	05/04/2011 13:18:54

MS Acq. : Proc. Methods	(Unspecified) : (Unspecified)
Interpretation Method	(Unspecified)

Gel Idx/Pos	339/N17	Instr./Gel Origin	Ak115/2011-03-18-1	Process Status	Analysis Succeeded
Plate [#] Name	[1] 001300010097	Instrument Sample Name		Spectra	6

Rank	Protein Name	Accession No.	Protein Score	Protein Score C. I. %	Total Ion Score	Total Ion C. I. %	Protein MW	Protein PI	Spot Name	Pep. Count	User name	Department
1	RecName: Full=Cytochrome b-c1 complex subunit 2, mitochondrial; AltName: Full=Complex III subunit 2	gi|14548302|sp|Q9DB77.1|QCR2_MOUSE	323	100	252	100	48262	9.26	H-H-4	11	Khalil	Medicinal Chemistry

Peptide Information

Calc. Mass	Obsrv. Mass	± da	± ppm	Start Seq.	End Seq.	Sequence	Ion Score	C. I. %	Modification	Rank	Result Type
1000.5686	1000.5305	-0.0381	-38	148	155	RWEVAALR					Mascot
1121.5698	1121.5382	-0.0316	-28	163	172	AVAFQNSQTR	60	99.996			Mascot
1235.6201	1235.5747	-0.0454	-37	232	241	QVAEQFLNMR					Mascot
1251.615	1251.5726	-0.0424	-34	232	241	QVAEQFLNMR	13	0	Oxidation (M)[9]		Mascot
1251.615	1251.5726	-0.0424	-34	232	241	QVAEQFLNMR			Oxidation (M)[9]		Mascot
1262.6528	1262.5868	-0.066	-52	7	17	AGSFSRFYSLK					Mascot
1282.6096	1282.5726	-0.037	-29	117	127	ENMAYTVEGIR					Mascot
1298.6045	1298.5636	-0.0409	-31	117	127	ENMAYTVEGIR			Oxidation (M)[3]		Mascot
1314.7052	1314.6622	-0.043	-33	173	183	IIENLHDVAYK					Mascot
1419.7438	1419.6941	-0.0497	-35	302	315	GNNTTSLLSQSVAK					Mascot
1477.7758	1477.7301	-0.0457	-31	160	172	IDKAVAFQNSQTR					Mascot
1529.7917	1529.7397	-0.052	-34	360	375	AVAQGNLSSADVQAAK	96	100			Mascot
1529.7917	1529.7397	-0.052	-34	360	375	AVAQGNLSSADVQAAK					Mascot
1618.782	1618.724	-0.058	-36	71	84	YEDSNNLGTSHLLR	83	100			Mascot
1618.782	1618.724	-0.058	-36	71	84	YEDSNNLGTSHLLR					Mascot
2179.0566	2178.9551	-0.1015	-47	200	217	ITSEELHYFVQNHFTSAR					Mascot

12. Acetyl-CoA acetyltransferase

Analysis Information

Report Type	Protein-Peptide Summary by Spot	Analysis Type	Combined (MS+MS/MS)
Sample Set Name	2011-05-04 [001300010097-2011-03-18-1]	Database	SwissProt
Analysis Name	Khalil-Medicinal Chemistry[Mus Swissprot]	Creation Date	05/04/2011 13:12:29
Reported By	05/04/2011 17:22:28 - admin	Last Modified	05/04/2011 13:18:54

MS Acq. : Proc. Methods	(Unspecified) : (Unspecified)
Interpretation Method	(Unspecified)

Gel Idx/Pos	333/N11	Instr./Gel Origin	Ak115/2011-03-18-1	Process Status	Analysis Succeeded
Plate [#] Name	[1] 001300010097	Instrument Sample Name		Spectra	6

Rank	Protein Name	Accession No.	Protein Score	Protein Score C. I. %	Total Ion Score	Total Ion C. I. %	Protein MW	Protein PI	Spot Name	Pep. Count	User name	Department
	acetyl-CoA acetyltransferase, mitochondrial precursor [Mus musculus]	gi|21450129	81	99.879	68	99.996	45129.5	8.71	H-H-4	4	Khalil	Medicinal Chemistry

Peptide Information

Calc. Mass	Obsrv. Mass	± da	± ppm	Start Seq.	End Seq.	Sequence	Ion Score	C. I. %	Modification	Rank	Result Type
910.5832	910.5497	-0.0335	-37	301	308	LNVKPLAR					Mascot
973.5023	973.4964	-0.0059	-6	363	370	MLEIDPQK					Mascot
1561.7129	1561.662	-0.0509	-33	206	218	QEQDTYALSSYTR	68	99.996			Mascot
1561.7129	1561.662	-0.0509	-33	206	218	QEQDTYALSSYTR					Mascot
2179.0085	2178.9551	-0.0534	-25	201	218	MNISRQEQDTYALSSYTR			Oxidation (M)[1]		Mascot

13. Cytochrome c1

Analysis Information

Report Type	Protein-Peptide Summary by Spot	Analysis Type	Combined (MS+MS/MS)
Sample Set Name	2011-05-04 [001300010097-2011-03-18-1]	Database	SwissProt
Analysis Name	Khalil-Medicinal Chemistry[Mus Swissprot]	Creation Date	05/04/2011 13:12:29
Reported By	05/04/2011 17:22:34 - admin	Last Modified	05/04/2011 13:18:54

MS Acq. : Proc. Methods	(Unspecified) : (Unspecified)
Interpretation Method	(Unspecified)

**Gel Idx/Pos**	342/N20	**Instr./Gel Origin**	Ak115/2011-03-18-1	**Process Status**	Analysis Succeeded
**Plate [#] Name**	[1] 001300010097	**Instrument Sample Name**		**Spectra**	6

Rank	Protein Name	Accession No.	Protein Score	Protein Score C. I. %	Total Ion Score	Total Ion C. I. %	Protein MW	Protein PI	Spot Name	Pep. Count	User name	Department
1	RecName: Full=Cytochrome c1, heme protein, mitochondrial; AltName: Full=Complex III subunit 4; AltN	gi|21759079|sp|Q9D0M3.1|CY1_MOUSE	277	100	252	100	35533	9.24	H-K-5	5	Khalil	Medicinal Chemistry

Peptide Information

Calc. Mass	Obsrv. Mass	± da	± ppm	Start Seq.	End Seq.	Sequence	Ion Score	C. I. %	Modification	Rank	Result Type
972.5989	972.5772	-0.0217	-22	318	325	KLAYRPPK					Mascot
1033.5571	1033.5402	-0.0169	-16	1	10	MAAAAASLRR			Oxidation (M)[1]		Mascot
1298.7063	1298.7196	0.0133	10	100	111	GLLSSLDHTSIR	62	99.998			Mascot
1298.7063	1298.7196	0.0133	10	100	111	GLLSSLDHTSIR					Mascot
1670.886	1670.8984	0.0124	7	187	202	AANNGALPPDLSYIVR	96	100			Mascot
1670.886	1670.8984	0.0124	7	187	202	AANNGALPPDLSYIVR					Mascot
1864.9229	1864.9381	0.0152	8	171	186	LSDYFPKPYPNPEAAR	93	100			Mascot
1864.9229	1864.9381	0.0152	8	171	186	LSDYFPKPYPNPEAAR					Mascot

14. Proteasome subunit alpha type-6

Analysis Information

Report Type	Protein-Peptide Summary by Spot	Analysis Type	Combined (MS+MS/MS)
Sample Set Name	2011-05-04 [001300010097-2011-03-18-1]	Database	SwissProt
Analysis Name	Khalil-Medicinal Chemistry[Mus Swissprot]	Creation Date	05/04/2011 13:12:29
Reported By	05/04/2011 17:22:34 - admin	Last Modified	05/04/2011 13:18:54

MS Acq. : Proc. Methods	(Unspecified) : (Unspecified)
Interpretation Method	(Unspecified)

**Gel Idx/Pos**	342/N20	**Instr./Gel Origin**	Ak115/2011-03-18-1	**Process Status**	Analysis Succeeded
**Plate [#] Name**	[1] 001300010097	**Instrument Sample Name**		**Spectra**	6

Rank	Protein Name	Accession No.	Protein Score	Protein Score C. I. %	Total Ion Score	Total Ion C. I. %	Protein MW	Protein PI	Spot Name	Pep. Count	User name	Department
[illegible]	RecName: Full=Proteasome subunit alpha type-6; AltName: Full=Macropain iota chain; AltName: Full=Mu	gi|9910829|sp|Q9QUM9.1|PSA6_MOUSE	170	100	147	100	27811	6.34	H-K-5	5	Khalil	Medicinal Chemistry

Peptide Information

Calc. Mass	Obsrv. Mass	± da	± ppm	Start Seq.	End Seq.	Sequence	Ion Score	C. I. %	Modification	Rank	Result Type
1033.5347	1033.5402	0.0055	5	46	54	DCAVIVTQK			Carbamidomethyl (C)[2]		Mascot
1156.611	1156.627	0.016	14	12	21	HITIFSPEGR	58	99.994			Mascot
1156.611	1156.627	0.016	14	12	21	HITIFSPEGR					Mascot
1170.532	1170.6147	0.0827	71	1	11	MSRGSSAGFDR					Mascot
1285.7223	1285.7378	0.0155	12	31	43	AINQGGLTSVAVR	90	100			Mascot
1285.7223	1285.7378	0.0155	12	31	43	AINQGGLTSVAVR					Mascot

15. Proteasome subunit alpha type-4

Analysis Information

Report Type	Protein-Peptide Summary by Spot	Analysis Type	Combined (MS+MS/MS)
Sample Set Name	2011-05-04 [001300010097-2011-03-18-1]	Database	SwissProt
Analysis Name	Khalil-Medicinal Chemistry[Mus Swissprot]	Creation Date	05/04/2011 13:12:29
Reported By	05/04/2011 17:22:35 - admin	Last Modified	05/04/2011 13:18:54

MS Acq. : Proc. Methods	(Unspecified) : (Unspecified)
Interpretation Method	(Unspecified)

**Gel Idx/Pos**	343/N21	**Instr./Gel Origin**	Ak115/2011-03-18-1	**Process Status**	Analysis Succeeded
**Plate [#] Name**	[1] 001300010097	**Instrument Sample Name**		**Spectra**	6

Rank	Protein Name	Accession No.	Protein Score	Protein Score C. I. %	Total Ion Score	Total Ion C. I. %	Protein MW	Protein PI	Spot Name	Pep. Count	User name	Department
1	RecName: Full=Proteasome subunit alpha type-4; AltName: Full=Macropain subunit C9; AltName: Full=Mu	gi|9910830|sp|Q9R1P0.1|PSA4_MOUSE	159	100	127	100	29737.3	7.59	H-K-6	6	Khalil	Medicinal Chemistry

Peptide Information

Calc. Mass	Obsrv. Mass	± da	± ppm	Start Seq.	End Seq.	Sequence	Ion Score	C. I. %	Modification	Rank	Result Type
902.5305	902.5261	-0.0044	-5	211	218	VEIATLTR					Mascot
999.4894	999.5169	0.0275	28	119	127	QAYTQFGGK					Mascot
1007.5156	1007.5313	0.0157	16	9	17	TTIFSPEGR	52	99.975			Mascot
1007.5156	1007.5313	0.0157	16	9	17	TTIFSPEGR					Mascot
1226.6304	1226.645	0.0146	12	55	64	LLDEVFFSEK					Mascot
1357.6304	1357.7034	0.073	54	177	187	QDYKEGEMTLK			Oxidation (M)[8]		Mascot
1430.8213	1430.8401	0.0188	13	206	218	LSAEKVEIATLTR	75	100			Mascot
1430.8213	1430.8401	0.0188	13	206	218	LSAEKVEIATLTR					Mascot
